# Nutritional Interventions for the Prevention of Cognitive Decline in Patients With Mild Cognitive Impairment and Alzheimer Disease: Protocol for a Network Meta-Analysis of Randomized Controlled Trials

**DOI:** 10.2196/47196

**Published:** 2024-02-28

**Authors:** Qian He, Kevin Chun Hei Wu, Adam N Bennett, Jia Yue Zhang, Kei Hang Katie Chan

**Affiliations:** 1 Department of Biomedical Sciences City University of Hong Kong Hong Kong China; 2 Department of Electrical Engineering City University of Hong Kong Hong Kong China; 3 Department of Epidemiology, Centre for Global Cardiometabolic Health Brown University Providence, RI United States

**Keywords:** network meta-analysis, cognitive impairment, Alzheimer disease, Alzheimer, neurodegenerative disorders, geriatric care, nutritional interventions, older patient, geriatric, cognitive decline, aging, older people, nutrition, cognitive, aging, intervention, Alzheimer disease, dementia, acute confusional senile dementia, Elder Nutritional Physiological Phenomena, Nutrition Physiology, nutrition, meta-analysis, meta-analyses, systematic review, systematic reviews

## Abstract

**Background:**

Mild cognitive impairment (MCI) is the stage between cognitive decline due to physiological aging and the severity of decline seen in neurodegenerative disorders like Alzheimer disease (AD), which is among the most prevalent neurodegenerative disorders characterized by cognitive impairment. People with MCI are at increased risk of developing AD. Although MCI and AD are incurable, nutritional interventions can potentially delay or prevent their onset. Consequently, effective interventions used to decelerate or alleviate the progress of cognitive impairment in older people are a significant focus in geriatric care. Given the synergistic effects of nutrition on health, assessing the effectiveness of nutritional supplements or dietary composition in preventing MCI or AD is essential for developing interventional strategies.

**Objective:**

Our study aims to assess the effectiveness of various nutritional interventions, including special dietary types, dietary patterns, specific foods, nutritional intake, and nutritional supplements, in preventing cognitive decline among patients diagnosed with MCI or AD. To achieve this, we will use a comprehensive approach, including network meta-analysis, pairwise meta-analysis, and systematic review of randomized controlled trials (RCTs).

**Methods:**

The review will follow the Population, Intervention, Comparison, Outcome (PICO) model and the PRISMA-P (Preferred Reporting Items for Systematic Review and Meta-Analysis Protocols) guidelines. Two investigators will independently search PubMed electronically. Data extraction will follow the inclusion criteria, and data will be assessed for risk of bias using a revised tool. Additionally, evidence quality will be evaluated using the Grading of Recommendations, Assessment, Development and Evaluation (GRADE) framework. The outcomes of interest are assessing the cognitive outcomes in patients with MCI or AD. A systematic literature search will be conducted, identifying randomized controlled trials that investigate the impact of these nutritional interventions on cognitive function decline in individuals with MCI and AD. Network meta-analyses (random-effects model) and pairwise meta-analyses will then estimate the relative effectiveness of different nutritional interventions.

**Results:**

We included 51 studies, published between 1999 and 2023 (27 studies for AD and 24 studies for MCI) and involving 8420 participants. We completed data extraction for all 51 studies by December 2023. Currently, we are actively engaged in data analysis and manuscript preparation. We plan to finalize the manuscript and publish the comprehensive results by the end of 2024.

**Conclusions:**

Our study holds significant clinical relevance given the rising prevalence of AD and the potential influence of nutritional interventions on cognitive function in individuals with MCI and AD. By investigating this relationship, our research aims to inform evidence-based decision-making in the development of prevention strategies for MCI and AD. The outcomes are expected to contribute to the establishment of reliable recommendations for MCI or AD management, providing substantial support in the field.

**Trial Registration:**

PROSPERO CRD42022331173; http://tinyurl.com/3snjp7a4

**International Registered Report Identifier (IRRID):**

PRR1-10.2196/47196

## Introduction

Cognitive decline, particularly associated with mild cognitive impairment (MCI) and Alzheimer disease (AD), represents a significant and growing public health concern worldwide [1]. As the aging population continues to increase, the prevalence of MCI and AD is also on the rise, leading to substantial personal, societal, and economic burdens [2]. One study indicated that the occurrence of MCI was higher than 22.5 per 1000 person-years among people aged 75 years and older [3]. Patients with MCI are also at a high risk of developing AD [4,5]. Therefore, identifying effective interventions to prevent or delay cognitive decline in individuals with MCI and AD is of utmost importance.

Currently, MCI and AD are incurable; however, delay and prevention of MCI or AD are possible [6]. Modifiable factors such as metabolic conditions (eg, diabetes), vascular issues (eg, hypertension), mental health concerns (eg, depression), social factors (eg, isolation), and lifestyle choices (eg, nutritional intake [7], dietary type [8], and physical inactivity) have been frequently linked with the risk of MCI and AD [9]. As such, it is critical to understand how modulating health and lifestyle factors may prevent cognitive decline. In recent decades, nutritional interventions have gained attention as potential strategies for cognitive decline prevention [10,11]. Various nutrients, dietary patterns, and dietary supplements have been investigated for their potential benefits in cognitive function and neuroprotection. Multiple types of intervention have been applied to these populations to reduce the decline of cognitive function, such as the Mediterranean diet and the ketogenic diet as well as the regulation of fatty acids, antioxidants, vitamins, and micronutrients [12-15]. Andrews et al [16] conducted a systematic review and meta-analysis of randomized controlled trials (RCTs) to evaluate the effect of dietary patterns, food, and nutritional supplements on cognitive function in individuals with MCI. The findings of this review demonstrated that few nutritional interventions convincingly improved the cognition of individuals with MCI [16]. Vlachos et al [17] conducted a systematic review and found folate, vitamin E, omega-3 fatty acids, and certain multinutrient formulations have shown some preliminary promising results [17]. However, the evidence regarding the effectiveness of different nutritional interventions remains inconclusive, with conflicting findings reported across individual RCTs.

To address the limitations of traditional pairwise meta-analyses and provide a comprehensive synthesis of the available evidence, we propose conducting a network meta-analysis (NMA). An NMA is a type of meta-analysis that extends beyond regular meta-analyses [18,19]. By using this approach, we aim to provide more precise and robust estimates of the effects of different nutritional interventions on cognitive decline in patients with MCI and AD. In this protocol, we outline the methodology for conducting an NMA of RCTs on nutritional interventions for the prevention of cognitive decline in patients with MCI and AD.

## Methods

### Experimental Approach

This protocol was developed following the PRISMA-P (Preferred Reporting Items for Systematic Review and Meta-Analysis Protocols) 2015 checklist [20]. This protocol has been registered with the PROSPERO database (CRD42022331173).

### Inclusion and Exclusion Criteria

The inclusion and exclusion criteria will be determined according to the principle of the Population, Intervention, Comparison, Outcome (PICO) design. The inclusion and exclusion criteria are presented briefly in Table 1.

**Table 1:** Inclusion and exclusion criteria for studies in the meta-analysis.

Variables	Inclusion criteria	Exclusion criteria
Study design	RCTs^a^ reported as comparing one nutritional intervention with another or a placebo	Non-RCTs
Participants	Participants with MCI^b^ or AD^c^ older than 50 years	Participants with vascular dementia; Parkinson disease–related dementia; delirium; depression and other mental illnesses; congenital brain function hypoplasia, such as Down syndrome; or subarachnoid hemorrhage
Diagnosis of MCI or AD	Diagnosis according to NINCDS-ADRDA^d^ DSM^e^ or assessed by MMSE^f^ with scores between 10 and 26	N/A^g^
Nutritional intervention assessment	Any type of nutritional intervention, such as special dietary type, dietary pattern, food, nutritional intake, and nutritional supplements at all doses or ingested amounts with a duration of more than 12 weeks	No definitive preventative impact was evaluated, or the effects were evaluated for less than 12 weeks.
Outcome measurement	The primary outcome will be assessed by the MMSE. The secondary outcomes will be assessed with other scales like the ADAS-Cog^h^, CDR^i^, and WAIS-R^j^ scores.	N/A

^a^RCTs: randomized controlled trials.

^b^MCI: mild cognitive impairment.

^c^AD: Alzheimer disease.

^d^NINCDS-ADRDA: National Institute of Neurological and Communicative Disorders and Stroke and the Alzheimer’s Disease and Related Disorders Association.

^e^DSM: Diagnostic and Statistical Manual of Mental Disorders.

^f^MMSE: Mini-Mental State Examination.

^g^N/A: not applicable.

^h^ADAS-Cog: Alzheimer’s Disease Assessment Scale cognitive subscale.

^i^CDR: Clinical Dementia Rating Scale.

^j^WAIS-R: Wechsler Adult Intelligence Scale-Revised.

### Inclusion Criteria

#### Type of Study

All RCTs that compared one nutritional intervention with another or a placebo for the prevention of cognitive decline in patients with MCI or AD will be included.

#### Participants

Participants with MCI or AD older than 50 years, regardless of gender, ethnicity or race, geography, dwelling, or chronic diseases, will be included in the analysis.

#### Inclusion of Patients With Different Degrees of MCI or AD

Participants will be considered as having AD by fulfilling the requirement of the National Institute of Neurological and Communicative Disorders and Stroke and the Alzheimer’s Disease and Related Disorders Association (NINCDS-ADRDA) or the Diagnostic and Statistical Manual of Mental Disorders (DSM) [21,22]. Participants will be evaluated for MCI using the Mini-Mental State Examination (MMSE) [23].

#### Nutritional Interventions Assessment

Experimental interventions included any type of nutritional intervention, such as special dietary type, dietary pattern, food, nutritional intake, and nutritional supplements at all doses or ingested amounts. Interventions lasted more than 12 weeks and were with or without medication as cointervention. The control group received a placebo intervention.

#### Outcome Measurements

Both the primary outcome and secondary outcomes involve the assessment of the cognitive function. The primary outcome will be assessed by the MMSE and the Alzheimer’s Disease Assessment Scale cognitive subscale (ADAS-Cog). The secondary outcomes will be assessed using other scales, such as the Clinical Dementia Rating Scale (CDR), and the Wechsler Adult Intelligence Scale-Revised (WAIS-R). The standard mean difference (SMD; Cohen *d*) will assess the effect sizes of the continuous variables. A small-sample (n<20) correction will be applied to SMD, leading to an effect size called Hedges *g*.

### Exclusion Criteria

The following studies were excluded:

- Studies were non-RCTs or in the form of a letter, editorial, commentary, or case report.
- There was no definitive preventative impact evaluated, or the effects were evaluated for less than 12 weeks.
- Participants had vascular dementia; Parkinson disease–related dementia; delirium; depression and other mental illnesses; congenital brain function hypoplasia, such as Down syndrome; or subarachnoid hemorrhage.

### Search Strategy

Two investigators (QH and KCHW) will independently perform the search strategy, and disagreements will be resolved by discussion or by a third investigator (JYZ). We will search for all the relevant citations published from the date of the respective database onset until Dec 31, 2023, in 3 English databases (ie, PubMed, Embase, and Cochrane Library). We will establish search strategies that combine keywords and indexed terms indicative of MCI or AD, nutritional intervention, and RCTs. Furthermore, the reference lists of the listed papers will be manually examined to seek new studies that are related. Any controversies will be resolved through dialogue. Multimedia Appendix 1 includes the search techniques.

### Data Extraction

Two investigators (QH and KCHW) will independently extract the data from all eligible studies published in English using standardized spreadsheets that are predefined. Any discrepancies will be resolved through consensus by discussion between the 2 investigators or, if necessary, arbitrated by a third investigator (JYZ). The following information will be extracted from every study: first author, publication year, detailed trial information (ie, randomization, sequence concealment, blinding, nutritional intervention protocols, number of treatment arms, as well as the dose and frequency of interventions), the diagnosis criteria, characteristics of the patient (eg, age, gender, race, and baseline MMSE or ADAS-Cog scores), sample size, follow-up MMSE or ADAS-Cog scores, number of completed interventions, as well as the duration of the intervention and follow-up. We will contact the authors via email to obtain the raw data when the data on outcomes are incomplete in the original article; otherwise, we will not include studies lacking such data. At last, 2 investigators will cross-check all retrieved data to verify their accuracy.

### Quality Assessment

The quality of the included studies will be evaluated by the Version 2 of the Cochrane risk-of-bias tool for randomized trials (RoB2) [24]. Various biases, such as selection bias (randomized sequence generation and allocation concealment), performance bias (blinding of participants and personnel), detection bias (blinding of outcome assessment), attrition bias (dealing with incomplete outcome data), reporting bias (selective reporting), and overall biases will be assessed in our study. The trials will be categorized into 3 types: low risk of bias, unclear risk of bias, and high risk of bias. Assessment of bias of the trials will be conducted by 2 investigators (QH and KCHW), and disagreements will be resolved through discussion with a third investigator (JYZ). Moreover, we will contact the authors asking for detailed allocation concealment and other characteristics when they are not available in the study.

### Statistical Synthesis

#### Characteristics of Included Studies and Information Flow in the Network

For the eligible trials included in our analysis, we will generate descriptive statistics to provide a comprehensive overview of the population characteristics and key variables across all trials. These descriptive statistics will encompass factors such as age, disease subtype, and other relevant risk factors that may impact the outcomes of interest. To visually represent the network of evidence, we will construct a network diagram. The edges connecting different interventions will be depicted with widths proportional to the inverse of the variance of the summary impact for each direct treatment comparison. The size of the nodes in the diagram will correspond to the quantity of evidence accumulated for each treatment, represented by the total number of studies contributing to that node. In addition, we will use a contribution matrix to explore the direct comparisons that have a substantial influence on the relative effects within the network [21,22]. This matrix will help identify the specific comparisons that contribute significantly to the overall findings.

#### Assessment of the Transitivity Assumption

The transitivity assumption will be assessed by evaluating the distribution of modifiable variables, such as age and mean baseline cognitive function, between the intervention and the control group [23].

#### Pairwise Meta-Analyses and Network Meta-Analyses

By conducting the pairwise meta-analysis, we can quantitatively assess the effectiveness of interventions that have direct evidence. This analysis allows for a comparison of the treatment effects within individual studies, providing valuable insights into the efficacy of each intervention. To ensure transparency and facilitate interpretation, we will present the results of the pairwise meta-analysis using forest plots.

The NMA will be conducted using a Bayesian framework, pooling data from all included studies [24]. To provide a visual representation of the network of evidence, we will generate network plots, which summarize the geometry of the network. These plots will help illustrate the connections between different interventions and highlight the available evidence for each comparison. For the estimation of effect sizes, we will use contrast-based methods within the NMA framework. The surface under the cumulative ranking curve will be used to estimate the relative rank of the different nutritional interventions [25].

All data will be analyzed using the R 4.0.3 (R Core Team) software, specifically using the gemtc package. Estimates will be reported as SMDs and 95% credible intervals (CrIs). We will use 3 Markov chains with 100,000 iterations after an initial thinning of 10,000 and a thinning of 10. The convergence of the model will be estimated by checking trace plots and the Brooks-Gelman-Rubin statistical value.

A systematic review will be considered when studies are scarce, typically involving less than 2 available studies. Additionally, if the heterogeneity observed among the included studies is exceptionally high, typically exceeding 95%, a systematic review approach will be adopted.

#### Assessment of Inconsistency for Network Meta-Analysis (NMA)

Consistency refers to the statistical manifestation of transitivity while a lack thereof is known as inconsistency [26]. Inconsistency suggests a disagreement between these estimates and indicates potential issues with the consistency of evidence across the network of studies. We will use the node-splitting method to assess inconsistency [27,28]. The presence of inconsistency between different estimates using direct and indirect evidence can be evaluated by assessing the *P* value. Inconsistency in the network is indicated by *P*<.05.

#### Assessment of Heterogeneity and Sensitivity Analyses

To quantify between-study heterogeneity, we will calculate the *I*^2^ statistics with 95% CrI and *τ*^2^ to convey the heterogeneity. *τ*^2^ represents the variability in intervention effects across studies, while *I*^2^ quantifies the proportion of total variability that is due to heterogeneity rather than chance. If *I*^2^ >50%, it will be considered as moderate to substantial heterogeneity; in such cases, the effect sizes will be estimated using a random-effects model [29]. Moreover, we will estimate the outlier and influential study when *I*^2^>50%, and then, we will perform the NMA or pairwise meta-analysis again, excluding the outliers and influential studies. Study-specific estimates and 95% CrIs, will be presented by forest plots. Subgroup meta-analyses and meta-regression analysis will be performed to estimate the source of heterogeneity. Subgroup analyses by different groups allow us to test specific hypotheses and describe why certain types of studies produce lower or higher effects than others. Performing subgroup analysis would help estimate if the interventional effects for the primary outcomes are robust. Meta-regression analysis could help quantify the effects by including covariates in the network meta-analysis models, if suitable. Then we will run subgroup and meta-regression with the following criteria: cognitive dysfunction severity at baseline, study year, sample size, risk of bias, duration of intervention, and number of recruiting centers (ie, single-center or multicentric studies).

Sensitivity analyses will be conducted to assess the robustness of the primary outcome results in the meta-analysis. These analyses will involve examining the impact of outliers, influential studies, risk of bias, and sample size by using the leave-one-out method. Specifically, each study will be systematically removed from the meta-analysis, and the pooled effect will be re-evaluated both in pairwise meta-analysis and NMA for each iteration. Studies with changes in the effect size exceeding 10% will be noted.

#### Assessment of Publication Bias

The comparison-adjusted [26] and contour-enhanced funnel plots [30] will be used to find out whether the results in imprecise trials differ from more precise trials. In addition, network meta-regression models and the Egger test will be performed to detect the effects between study size and effect size and to assess the asymmetry of the funnel plot, respectively.

## Results

The initial search yielded 30,269 citations, of which 248 articles were identified as potentially eligible and will undergo evaluation for eligibility. Among these, a total of 197 articles were excluded for various reasons. This exclusion consisted of 3 duplicates, 39 articles without full-text availability, 45 articles lacking an assessment of cognitive function, 40 articles not meeting the criteria of being RCTs, 28 articles not in English, and 42 articles unrelated to the study objective. Ultimately, 51 studies meeting the inclusion criteria were included in our NMA. These studies, published between 1999 and 2023, consisted of 27 studies focusing on AD and 24 studies focusing on MCI. In total, these studies involved 8420 participants. We completed data extraction for all 51 studies by December 2023. Currently, we are actively engaged in data analysis and manuscript preparation. We plan to finalize the manuscript and publish the comprehensive results by the end of 2024.

Ethical approval was not required in the meta-analysis. Our study is merely based on the published literature, for which ethical approval has been obtained. To disseminate the evidence obtained, we will publish our results in an international peer-reviewed journal to improve preventive applications with scientific evidence.

## Discussion

The global phenomenon of population aging has led to a significant increase in the prevalence of cognitive impairment, particularly AD and MCI [31]. This demographic shift has garnered substantial attention from health care providers, researchers, and policy makers due to the immense personal, societal, and economic implications [32]. As the current treatments for MCI and AD are limited and primarily focused on symptom management, identifying effective preventive strategies is of paramount importance [15]. The potential benefits of nutritional interventions for cognitive health have been a subject of considerable interest [2]. Various nutrients, dietary patterns, and dietary supplements have been investigated for their potential neuroprotective effects and cognitive function improvement. Therefore, through this study, we aim to consolidate the available evidence and provide a rigorous and comprehensive synthesis using the NMA approach. Moreover, identifying the most effective interventions will help guide future research efforts and resource allocation toward the most promising strategies.

It is important to acknowledge the limitations of this study. Although an NMA provides a robust statistical framework for synthesizing evidence, it is reliant on the quality and availability of data from included RCTs. Furthermore, potential sources of bias and heterogeneity among the studies may affect the robustness of the results.

In conclusion, the findings of this study will have significant implications for clinical practices as well as for informing public health strategies and policy making.

## Appendix

Multimedia Appendix 1 Search techniques.

## Abbreviations

AD: Alzheimer disease
ADAS-Cog: Alzheimer’s Disease Assessment Scale cognitive subscale
CDR: Clinical Dementia Rating Scale
CrI: credible interval
DSM: Diagnostic and Statistical Manual of Mental Disorders
GRADE: Grading of Recommendations, Assessment, Development and Evaluation
MCI: mild cognitive impairment
MMSE: Mini-Mental State Examination
NINCDS-ADRDA: National Institute of Neurological and Communicative Disorders and Stroke and the Alzheimer’s Disease and Related Disorders Association
NMA: network meta-analysis
PICO: Population, Intervention, Comparison, Outcome
PRISMA-P: Preferred Reporting Items for Systematic Review and Meta-Analysis Protocols
RCT: randomized controlled trial
SMD: standard mean difference
WAIS-R: Wechsler Adult Intelligence Scale-Revised
