# Supplementary material for: Nutritional Interventions for the Prevention of Cognitive Decline in Patients With Mild Cognitive Impairment and Alzheimer Disease: Protocol for a Network Meta-Analysis of Randomized Controlled Trials
Source: JMIR Res Protoc. 2024 Feb 28;13:e47196. doi: 10.2196/47196 (PMC10938226; doi:10.2196/47196)
Supplement: Multimedia Appendix 1 [file resprot_v13i1e47196_app1.pdf]

Supplementary 1 Relevant databases retrieval record

Search	Query
#143	Search #36 AND #45 AND #66 AND #142
#142	Search #91 OR #104 OR #141
#141	Search #105 OR #106 OR #107 OR #108 OR #109 OR #110 OR #111 OR #112 OR #113 OR #114 OR #115 OR #116 OR #117 OR #118 OR #119 OR #120 OR #121
#140	Search "ad libitum diets"[Title/Abstract]
#139	Search "standard dietary recommendations"[Title/Abstract]
#138	Search "diabetes prevention-type diets"[Title/Abstract]
#137	Search "energy-restricted diets"[Title/Abstract]
#136	Search "low fat diet"[Title/Abstract]
#135	Search "The American Heart Association Diet (AHAD)"[Title/Abstract]
#134	Search "Mediterranean-style ketogenic diet (MMKD)" [Supplementary Concept]
#132	Search Multicultural Healthy Diet (MHD) diet[Title/Abstract]
#132	Search "Modified Mediterranean-ketogenic diet (MMK)"[Title/Abstract]
#131	Search "low sodium"[Title/Abstract]
#130	Search "Atkins"[Title/Abstract]
#129	Search low-calorie[Title/Abstract]
#128	Search Palaeolithic[Title/Abstract]
#127	Search low glycaemic load[Title/Abstract]
#126	Search "low glycaemic index"[Title/Abstract]
#125	Search "vegan"[Title/Abstract]
#124	Search "vegetarian"[Title/Abstract]
#123	Search "high protein"[Title/Abstract]

#122	Search "low protein"[Title/Abstract]
#121	Search "high fat"[Title/Abstract]
#120	Search "low fat"[Title/Abstract]
#119	Search "high carbohydrate"[Title/Abstract]
#118	Search "low carbohydrate"[Title/Abstract]
#117	Search "Calorie Restriction (CR)"[Title/Abstract]
#116	Search "Atkins"[Title/Abstract]
#115	Search "MAD diet"[Title/Abstract]
#114	Search "modified Atkins diet"[Title/Abstract]
#113	Search "MCT diet" [Title/Abstract]
#112	Search "1medium chain triglyceride diet"[Title/Abstract]
#111	Search "Ketogenic diet"[Title/Abstract]
#110	Search "MIND diet"[Title/Abstract]
#109	Search "the Mediterranean DASH diet Intervention for Neurological Delay (MIND) diet "[Title/Abstract]
#108	Search"DASH" [Title/Abstract]
#107	Search"MeDi diet" [Title/Abstract]
#106	Search"MD diet" [Title/Abstract]
#105	Search"Mediterranean diet" [Title/Abstract]
#104	Search #92 OR #93 OR #94 OR #95 OR #96 OR #97 OR #98 OR #99 OR #100 OR #101 OR #102 OR #103
#103	Search "Diet, Reducing" [MeSH]
#102	Search "Diet, Sodium-Restricted" [MeSH]
#101	Search "Diet, Vegetarian" [MeSH]
#100	Search "Diet, Macrobiotic" [MeSH]
#99	Search "Diet, Fat-Restricted" [MeSH]

#98	Search "Diet, Protein-Restricted" [MeSH]
#97	Search "Diet, Mediterranean" [MeSH]
#96	Search "Diet, Carbohydrate-Restricted" [MeSH]
#95	Search "Diet, High-Fat" [MeSH]
#94	Search "Diet, Palaeolithic" [MeSH]
#93	Search "Diet, Western"[MeSH]
#92	Search "Diet"[MeSH]
#91	Search #67 OR #68 OR #69 OR #70 OR #71 OR #72 OR #73 OR #74 OR #75 OR #76 OR #77 OR #78 OR #79 OR #80 OR #81 OR #82 OR #83 OR #84 OR #85 OR #86 OR #87 OR #88 OR #89 OR #90
#90	Search "antioxidant"[Title/Abstract]
#89	Search "calcium"[Title/Abstract]
#88	Search "selenium"[Title/Abstract]
#87	Search "zinc"[Title/Abstract]
#86	Search "Cooper"[Title/Abstract]
#85	Search "Tocopherol"[Title/Abstract]
#84	Search "folate"[Title/Abstract]
#83	Search "vitamin C"[Title/Abstract]
#82	Search "vitamin E"[Title/Abstract]
#81	Search "vitamin A"[Title/Abstract]
#80	Search "vitamin B"[Title/Abstract]
#79	Search "vitamin"[Title/Abstract]
#78	Search "n-3"[Title/Abstract]
#77	Search "fatty acid"[Title/Abstract]
#76	Search "omega-3"[Title/Abstract]

#75	Search "docosahexaenoic acid"[Title/Abstract]
#74	Search "DHA"[Title/Abstract]
#73	Search "eicosapentaenoic acid"[Title/Abstract]
#72	Search "EPA"[Title/Abstract]
#71	Search "multi-vitamins"[Title/Abstract]
#70	Search "mineral"[Title/Abstract]
#69	Search "micronutrients supplementation "[Title/Abstract]
#68	Search "nutrition"[MeSH]
#67	Search "nutrient"[MeSH]
#66	Search #46 OR #47 OR #48 OR #49 OR #50 OR #51 OR #52 OR #53 OR #54 OR #55 OR #56 OR #57 OR #58 OR #59 OR #60 OR #61 OR #62 OR #63 OR #64 OR #65
#65	Search tripleblind*[Title/Abstract]
#64	Search trebleblind*[Title/Abstract]
#63	Search doubleblind*[Title/Abstract]
#62	Search singleblind*[Title/Abstract]
#61	Search blind*[Title/Abstract]
#60	Search random*[Title/Abstract]
#59	Search "Double-Blind Method"[MeSH]
#58	Search "Single-Blind Method"[MeSH]
#57	Search "Pragmatic Clinical Trials as Topic"[Publication Type]
#56	Search "Randomized Controlled Trials"[Publication Type]
#55	Search "Controlled Clinical Trials"[Publication Type]
#54	Search "Clinical Trials, Phase IV"[Publication Type]
#53	Search "Clinical Trials, Phase III"[Publication Type]

#52	Search "Clinical Trials, Phase II"[Publication Type]
#51	Search "Pragmatic Clinical Trials as Topic"[MeSH]
#50	Search "Intention to Treat Analysis"[MeSH]
#49	Search "Randomized Controlled Trials as Topic"[MeSH]
#48	Search "Clinical Trials, Phase IV as Topic"[MeSH]
#47	Search "Clinical Trials, Phase III as Topic"[MeSH]
#46	Search "Clinical Trials, Phase II as Topic"[MeSH]
#45	Search #36 OR #44
#44	Search #37 OR #38 OR #39 OR #40 OR #41 OR #42 OR #43
#43	Search "preclinical Alzheimer's disease"[Title/Abstract]
#42	Search "early onset Alzheimer's disease"[Title/Abstract]
#41	Search "early cognitive decline"[Title/Abstract]
#40	Search "pre-Alzheimer's disease"[Title/Abstract]
#39	Search "MCI"[Title/Abstract]
#38	Search "Search "mild cognitive impairment"[Title/Abstract]
#37	Search "Cognitive Dysfunction/epidemiology"[MeSH]
#36	Search #1 OR #2 OR #3 OR #4 OR #5 OR #6 OR #7 OR #8 OR #9 OR #10 OR #11 OR #12 OR #13 OR #14 OR #15 OR #16 OR #17 OR #18 OR #19 OR #20 OR #21 OR #22 OR #23 OR #24 OR #25 OR #26 OR #27 OR #28 OR #29 OR #30 OR #31 OR #32 OR #33 OR #34 OR #35
#35	Search "Presenile Alzheimer Dementia"[Title/Abstract]
#34	Search "Early Onset Alzheimer Disease"[Title/Abstract]
#33	Search "Alzheimer Disease, Early Onset"[Title]
#32	Search "Familial Alzheimer Diseases (FAD)"[Title/Abstract]
#31	Search "Alzheimer Diseases, Familial (FAD)"[Title/Abstract]
#30	Search "Alzheimer Disease, Familial (FAD)"[Title/Abstract]

#29	Search "Familial Alzheimer Disease (FAD)"[Title/Abstract]
#28	Search "Focal Onset Alzheimer's Disease"[Title/Abstract]
#27	Search "Alzheimer's Disease, Focal Onset"[Title/Abstract]
#26	Search "Late Onset Alzheimer Disease"[Title/Abstract]
#25	Search "Alzheimer Disease, Late Onset"[Title/Abstract]
#24	Search "Presenile Dementia"[Title/Abstract]
#23	Search "Dementia, Presenile"[Title/Abstract]
#22	Search "Senile Dementia, Acute Confusional"[Title/Abstract]
#21	Search "Acute Confusional Senile Dementia"[Title/Abstract]
#20	Search "Senile Dementia, Alzheimer Type"[Title/Abstract]
#19	Search "Dementias, Alzheimer"[Title/Abstract]
#18	Search "Dementia, Alzheimer"[Title/Abstract]
#17	Search "Alzheimer Dementias"[Title/Abstract]
#16	Search "Alzheimer Dementia"[Title/Abstract]
#15	Search "Alzheimer Syndrome"[Title/Abstract]
#14	Search "Sclerosis, Alzheimer"[Title/Abstract]
#13	Search "Alzheimer Sclerosis"[Title/Abstract]
#12	Search "Dementia, Primary Senile Degenerative"[Title/Abstract]
#11	Search "Primary Senile Degenerative Dementia"[Title/Abstract]
#10	Search "Alzheimer Type Senile Dementia"[Title/Abstract]
#9	Search "Dementia, Alzheimer-Type (ATD)"[Title/Abstract]
#8	Search "Alzheimer Type Dementia (ATD)"[Title/Abstract]
#7	Search "Alzheimer-Type Dementia (ATD)"[Title/Abstract]
#6	Search "Alzheimer Type Dementia"[Title/Abstract]

#5	Search "Dementia, Alzheimer Type"[Title/Abstract]
#4	Search "Senile Dementia"[Title/Abstract]
#3	Search "Dementia, Senile"[Title/Abstract]
#2	Search "Alzheimer's Disease"[Title/Abstract]
#1	Search "Alzheimer Disease"[MeSH]
